# Supplementary material for: A Uniform Benchmark for Testing SsrA-Derived Degrons in the Escherichia coli ClpXP Degradation Pathway
Source: Molecules. 2021 Sep 30;26(19):5936. doi: 10.3390/molecules26195936 (PMC8512704; doi:10.3390/molecules26195936)
Supplement: Supplementary file 1 [file molecules-26-05936-s001.zip › molecules-1396538-supplementary/Supplemetary Figures.pdf]

Supplemetary material

A uniform benchmark for testing ssrA-derived degrons in the Escherichia coli ClpXP degradation pathway

Maria Magdalena Klimecka, Anna Antosiewicz, Matylda Anna Izert, Patrycja Emanuela Szybowska, Piotr Krzysztof Twardowski, Clara Delaunay and Maria Wiktoria Górna

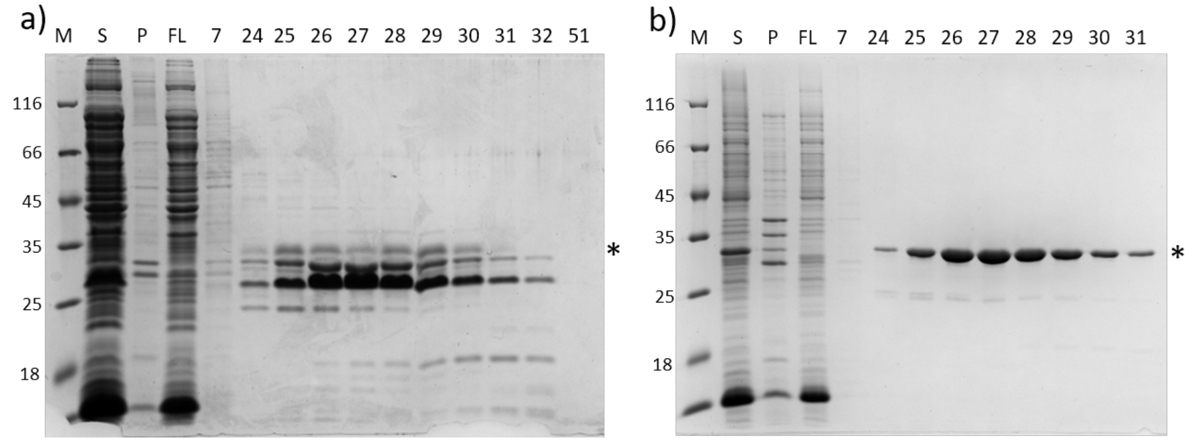

Supplementary Figure S1: Comparison of purification of eGFP–AANDENYSENY between proteins expressed in (**a**) TOP10 and (**b**) *ΔclpP E. coli* strains using tandem configuration of HisTrap and Superdex200 columns. M – molecular weight marker (kDa); S – the soluble fraction of the bacterial lysate prior to loading on the HisTrap column; P – the insoluble fraction (pellet) of the lysate ; FL – flow-through from the HisTrap column; 7-51 – individual fractions eluted from the Superdex200 column; * – intact, full length eGFP-AANDENYSENY protein. SDS-PAGE gels were stained with Coomassie.

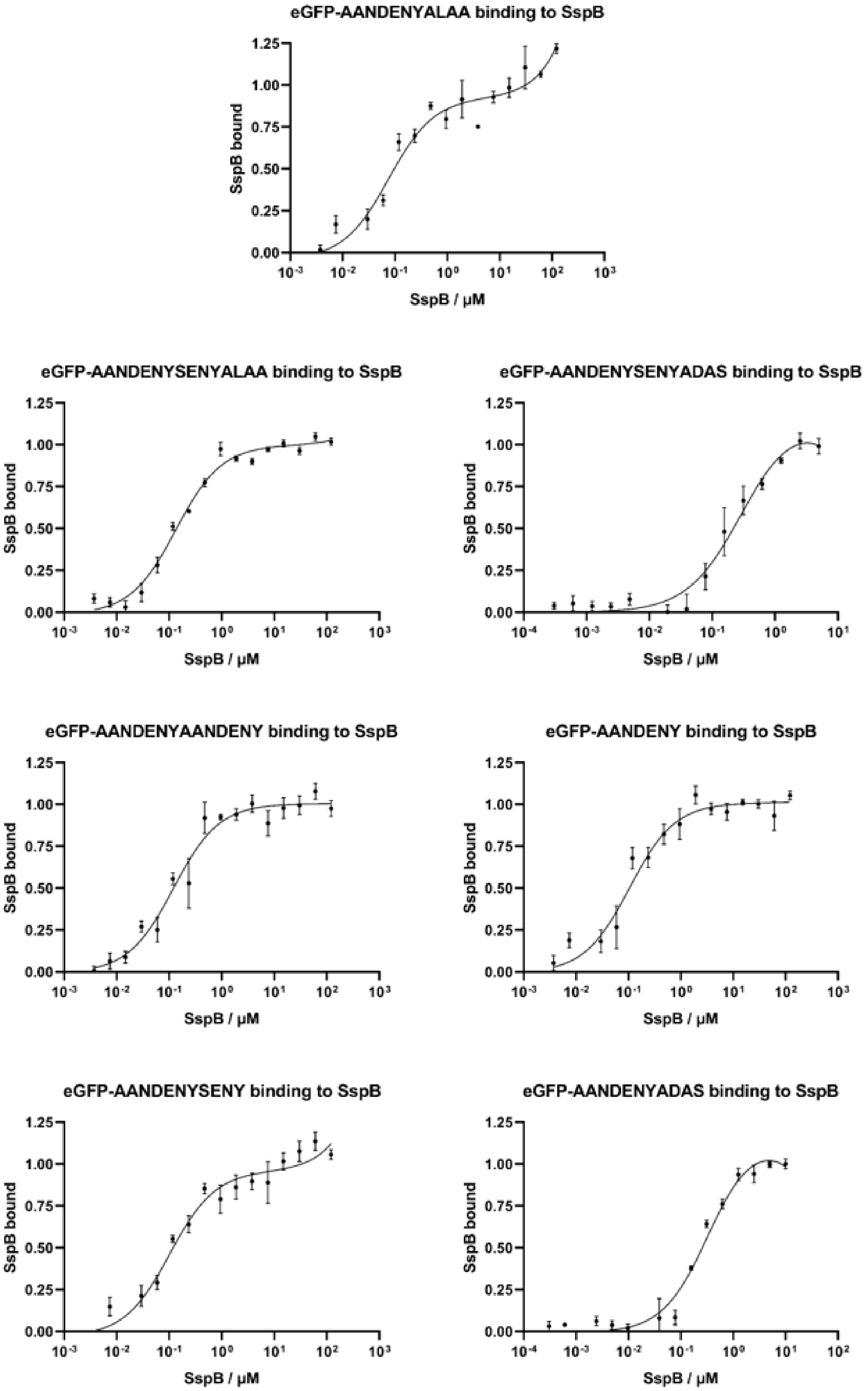

Supplementary Figure S2: MST results for the binding of eGFP-degrons containing the AANDENY motif to SspB. (**a**) eGFP-AANDENYALAA; (**b**) eGFP-AANDENYSENYALAA; (**c**) eGFP-AANDENYSENYADAS; (**d**) eGFP-AANDENYAANDENY; (**e**) eGFP-AANDENY; (**f**) eGFP-AANDENYENY; (**g**) eGFP-AANDENYADAS
