# Supplementary material for: A Uniform Benchmark for Testing SsrA-Derived Degrons in the Escherichia coli ClpXP Degradation Pathway
Source: Molecules. 2021 Sep 30;26(19):5936. doi: 10.3390/molecules26195936 (PMC8512704; doi:10.3390/molecules26195936)
Supplement: Supplementary file 1 [file molecules-26-05936-s001.zip › molecules-1396538-supplementary/Supplemetary Table S1.pdf]

Supplemetary material

A uniform benchmark for testing ssrA-derived degrons in the Escherichia coli ClpXP degradation pathway

Maria Magdalena Klimecka, Anna Antosiewicz, Matylda Anna Izert, Patrycja Emanuela Szybowska, Piotr Krzysztof Twardowski, Clara Delaunay and Maria Wiktoria Górna

Supplemetary Table S1: Sequences of the vectors used in the experiments

	Construct name	Nucleotide sequence of expression vector insert
1	pET28a/pBAD-ClpX	ATGGGCAGCAGCCATCATCATCATCATCACAGCAGCGGCCTGGTGCCGCGCGGCAGC<br>CATATGGAAGGTGAATATATTAAACTCAAAGTCATTGGACAGGATAGCAGTGAGATT<br>CACTTCAAAGTGAAAATGACAACACATCTCAAGAAACTCAAAGAATCATACTGTCAA<br>AGACAGGGTGTTCCAATGAATTCACTCAGGTTTCTCTTTGAGGGTCAGAGAATTGCT<br>GATAATCATACTCCAAAAGAACTGGGAATGGAGGAAGAAGATGTGATTGAAGTCTAC<br>CAGGAACAAACCGGTGGATCCATGACAGATAAACGCAAAGATGGCTCAGGCAAATTG<br>CTGTATTGCTCTTTTTGCGGCAAAAGCCAGCATGAAGTGCGCAAGCTGATTGCCGGT<br>CCATCCGTGTATATCTGCGACGAATGTGTTGATTTATGTAACGACATCATTCGCGAA<br>GAGATTAAAGAAGTTCACCGCATCGGAACCAGTCTACCGACGCCGCATGAAATTCGC<br>AACACCTGGACGATTACGTTTCGGCCAGGAACAGGCGAAAAAGGCTGGCGGTCCGG<br>TATACAACCATTACAAACGTTTGGCAACGGCGATACCAGCAATGCGTCGAGTTGGGC<br>AAAAGTAACATTCTGCTGATCGGTCCGACCGGTTCCGGTAAAACGCTGCTGGCTGAA<br>ACGCTGGCGCGCCTGCTGGATGTTCCGTTCACCATGGCCGACGCGACTACACTGACC<br>GAAGCCGGTTATGTGGGTGAAGACGTTGAAAACATCATTCAGAAGCTGTTGCAGAAA<br>TGCGACTACGATGTCCAGAAAGCACAGCGTGGTATTGTCTACATCGATGAAATCGAC<br>AAGATTTCTCGTAAGTCAGACAACCCGTCCATTACCCGAGACGTTTCCGGTGAAGGC<br>GTACAGCAGGCACTGTTGAAACTGATCGAAGGTACGGTAGCTGCTGTTCCACCGCAA<br>GGTGGGCGTAAACATCCGCAGCAGGAATTCTTGCAGGTTGATACCTCTAAGATCCTG<br>TTTATTTGTGGCGGTGCGTTTGCCGGTCTGGATAAAGTGATTTCCCACCGTGTAGAA<br>ACCGGCTCCGGCATTGGTTTTGGCGCGACGGTAAAAGCGAAGTCCGACAAAGCAAGC<br>GAAGGCGAGCTGCTGGCGCAGGTTGAACCGGAAGATCTGATCAAGTTTGGTCTTATC<br>CCTGAGTTTATTGGTCGTCTGCCGGTTGTCGCAACGTTGAATGAACTGAGCGAAGAA<br>GCTCTGATTCAGATCCTCAAAGAGCCGAAAAACGCCCTGACCAAGCAGTATCAGGCG<br>CTGTTTAATCTGGAAGGCGTGGATCTGGAATTCCGTGACGAGGCGCTGGATGCTATC<br>GCTAAGAAAGCGATGGCGCGTAAAACCGGTGCCCGTGGCCTGCGTCCCATCGTAGAA<br>GCCGCACTGCTCGATACCATGTACGATCTGCCGTCCATGGAAGACGTCGAAAAAGTG<br>GTTATCGACGAGTCGGTAATTGATGGTCAAAGCAAACCGTTGCTGATTTATGGCAAG<br>CCGGAAGCGCAACAGGCATCTGGTGAATAA
2	pET28a-ClpP	ATGGGCAGCAGCCATCATCATCATCATCACAGCAGCGGCCTGGTGCCGCGCGGCAGC<br>CATATGGAAGGTGAATATATTAAACTCAAAGTCATTGGACAGGATAGCAGTGAGATT<br>CACTTCAAAGTGAAAATGACAACACATCTCAAGAAACTCAAAGAATCATACTGTCAA<br>AGACAGGGTGTTCCAATGAATTCACTCAGGTTTCTCTTTGAGGGTCAGAGAATTGCT<br>GATAATCATACTCCAAAAGAACTGGGAATGGAGGAAGAAGATGTGATTGAAGTCTAC<br>CAGGAACAAACCGGTGGATCCATGTCATACAGCGGCGAACGAGATAACTTTGCACCC<br>CATATGGCGCTGGTGCCGATGGTCATTGAACAGACCTCACGCGGTGAGCGCTCTTTT<br>GATATCTATTCTCGTCTACTTAAGGAACGCGTCATTTTTCTGACTGGCCAGGTTGAA<br>GACCACATGGCTAACCTGATTGTGGCGCAGATGCTGTTCCTGGAAGCGGAAAACCCA<br>GAAAAAGATATCTATCTGTACATTAACTCCCCAGGCGGGGTGATCACTGCCGGGATG<br>TCTATCTATGACACCATGCAGTTTATCAAGCCTGATGTCAGCACCATCTGTATGGGC<br>CAGGCGGCCTCGATGGGCGCTTTCTTGCTGACCGCAGGGGCAAAAGGTAAACGTTTT<br>TGCCTGCCGAATTCGCGCGTGATGATTCACCAACCGTTGGGCGGCTACCAGGGCCAG<br>GCGACCGATATCGAAATTCATGCCCGTGAAATTCTGAAAGTTAAAGGGCGCATGAAT<br>GAACTTATGGCGCTTCATACGGGTCAATCATTAGAACAGATTGAACGTGATACCGAG

		CGCGATCGCTTCCTTTCCGCCCCTGAAGCGGTGGAATACGGTCTGGTCGATTCGATT<br>CTGACCCATCGTAATTGA
3	pET28a-SspB	ATGGGCAGCAGCCATCATCATCATCATCACAGCAGCGGCCTGGTGCCGCGCGGCAGC<br>CATATGGAAGGTGAATATATTAAACTCAAAGTCATTGGACAGGATAGCAGTGAGATT<br>CACTTCAAAGTGAAAATGACAACACATCTCAAGAAACTCAAAGAATCATACTGTCAA<br>AGACAGGGTGTTCCAATGAATTCACTCAGGTTTCTCTTTGAGGGTCAGAGAATTGCT<br>GATAATCATACTCCAAAAGAACTGGGAATGGAGGAAGAAGATGTGATTGAAGTCTAC<br>CAGGAACAAACCGGTGGATCCATGGATTTGTCACAGCTAACACCACGTCGTCCCTAT<br>CTGCTGCGTGCATTCTATGAGTGGTTGCTGGATAACCAGCTCACGCCGCACCTGGTG<br>GTGGATGTGACGCTCCCTGGCGTGCAGGTTCCTATGGAATATGCGCGTGACGGGCAA<br>ATCGTACTCAACATTGCGCCGCGTGCTGTCGGCAATCTGGAACTGGCGAATGATGAG<br>GTGCGCTTTAACGCGCGCTTTGGTGGCATTCCGCGTCAGGTTTCTGTGCCGCTGGCT<br>GCCGTGCTGGCTATCTACGCCCGTGAAAATGGCGCAGGCACGATGTTTGAGCCTGAA<br>GCTGCCTACGATGAAGATACCAGCATCATGAATGATGAAGAGGCATCGGCAGACAAC<br>GAAACCGTTATGTCGGTTATTGATGGCGACAAGCCAGATCACGATGATGACACTCAT<br>CCTGACGATGAACCTCCGCAGCCACCACGCGGTGGTCGACCGGCATTACGCGTTGTG<br>AAGTAACTCGAGCACCACCACCACCACCACTGAGATCCGGCTGCTAACAAAGCCCGA<br>AAGGAAGCTGAGTTGGCTGCTGCCACCGCTGAGCAATAACTAGCATAA
4	pBAD-eGFP-AANDENYALAA	ATGCGGGGTTCTCATCATCATCATCATCATGGTATGGCTAGCATGACTGGTGGACAG<br>CAAATGGGTCGGGATCTGTACGAGAACCTGTACTTCCAGGGCTCGAGCATGGTGAGC<br>AAGGGCGAGGAGCTGTTCACCGGGGTGGTGCCCATCCTGGTCGAGCTGGACGGCGAC<br>GTAAACGGCCACAAGTTCAGCGTGTCCGGCGAGGGCGAGGGCGATGCCACCTACGGC<br>AAGCTGACCCTGAAGTTCATCTGCACCACCGGCAAGCTGCCCGTGCCCTGGCCCACC<br>CTCGTGACCACCCTGACCTACGGCGTGCAGTGCTTCAGCCGCTACCCCGACCACATG<br>AAGCAGCACGACTTCTTCAAGTCCGCCATGCCCGAAGGCTACGTCCAGGAGCGCACC<br>ATCTTCTTCAAGGACGACGGCAACTACAAGACCCGCGCCGAGGTGAAGTTCGAGGGC<br>GACACCCTGGTGAACCGCATCGAGCTGAAGGGCATCGACTTCAAGGAGGACGGCAAC<br>ATCCTGGGGCACAAGCTGGAGTACAACTACAACAGCCACAACGTCTATATCATGGCC<br>GACAAGCAGAAGAACGGCATCAAGGTGAACTTCAAGATCCGCCACAACATCGAGGAC<br>GGCAGCGTGCAGCTCGCCGACCACTACCAGCAGAACACCCCCATCGGCGACGGCCCC<br>GTGCTGCTGCCCGACAACCACTACCTGAGCACCCAGTCCGCCCTGAGCAAAGACCCC<br>AACGAGAAGCGCGATCACATGGTCCTGCTGGAGTTCGTGACCGCCGCCGGGATCACT<br>CTCGGCATGGACGAGCTGTACAAGGCTGCAAACGACGAAAACTACGCTTTAGCAGCT<br>TAA
5	pBAD-eGFP-AANDENYSENYALAA	ATGCGGGGTTCTCATCATCATCATCATCATGGTATGGCTAGCATGACTGGTGGACAG<br>CAAATGGGTCGGGATCTGTACGAGAACCTGTACTTCCAGGGCTCGAGCATGGTGAGC<br>AAGGGCGAGGAGCTGTTCACCGGGGTGGTGCCCATCCTGGTCGAGCTGGACGGCGAC<br>GTAAACGGCCACAAGTTCAGCGTGTCCGGCGAGGGCGAGGGCGATGCCACCTACGGC<br>AAGCTGACCCTGAAGTTCATCTGCACCACCGGCAAGCTGCCCGTGCCCTGGCCCACC<br>CTCGTGACCACCCTGACCTACGGCGTGCAGTGCTTCAGCCGCTACCCCGACCACATG<br>AAGCAGCACGACTTCTTCAAGTCCGCCATGCCCGAAGGCTACGTCCAGGAGCGCACC<br>ATCTTCTTCAAGGACGACGGCAACTACAAGACCCGCGCCGAGGTGAAGTTCGAGGGC<br>GACACCCTGGTGAACCGCATCGAGCTGAAGGGCATCGACTTCAAGGAGGACGGCAAC<br>ATCCTGGGGCACAAGCTGGAGTACAACTACAACAGCCACAACGTCTATATCATGGCC<br>GACAAGCAGAAGAACGGCATCAAGGTGAACTTCAAGATCCGCCACAACATCGAGGAC<br>GGCAGCGTGCAGCTCGCCGACCACTACCAGCAGAACACCCCCATCGGCGACGGCCCC<br>GTGCTGCTGCCCGACAACCACTACCTGAGCACCCAGTCCGCCCTGAGCAAAGACCCC<br>AACGAGAAGCGCGATCACATGGTCCTGCTGGAGTTCGTGACCGCCGCCGGGATCACT<br>CTCGGCATGGACGAGCTGTACAAGGCTGCAAACGACGAAAACTACAGCGAAAACTAC<br>GCTTTAGCAGCTTAA
6	pBAD-eGFP-AANDENYSENYADAS	ATGCGGGGTTCTCATCATCATCATCATCATGGTATGGCTAGCATGACTGGTGGACAG<br>CAAATGGGTCGGGATCTGTACGAGAACCTGTACTTCCAGGGCTCGAGCATGGTGAGC<br>AAGGGCGAGGAGCTGTTCACCGGGGTGGTGCCCATCCTGGTCGAGCTGGACGGCGAC

		GTAAACGGCCACAAGTTCAGCGTGTCCGGCGAGGGCGAGGGCGATGCCACCTACGGC<br>AAGCTGACCCTGAAGTTCATCTGCACCACCGGCAAGCTGCCCGTGCCCTGGCCCACC<br>CTCGTGACCACCCTGACCTACGGCGTGCAGTGCTTCAGCCGCTACCCCGACCACATG<br>AAGCAGCACGACTTCTTCAAGTCCGCCATGCCCGAAGGCTACGTCCAGGAGCGCACC<br>ATCTTCTTCAAGGACGACGGCAACTACAAGACCCGCGCCGAGGTGAAGTTCGAGGGC<br>GACACCCTGGTGAACCGCATCGAGCTGAAGGGCATCGACTTCAAGGAGGACGGCAAC<br>ATCCTGGGGCACAAGCTGGAGTACAACTACAACAGCCACAACGTCTATATCATGGCC<br>GACAAGCAGAAGAACGGCATCAAGGTGAACTTCAAGATCCGCCACAACATCGAGGAC<br>GGCAGCGTGCAGCTCGCCGACCACTACCAGCAGAACACCCCCATCGGCGACGGCCCC<br>GTGCTGCTGCCCGACAACCACTACCTGAGCACCCAGTCCGCCCTGAGCAAAGACCCC<br>AACGAGAAGCGCGATCACATGGTCCTGCTGGAGTTCGTGACCGCCGCCGGGATCACT<br>CTCGGCATGGACGAGCTGTACAAGGCTGCAAACGACGAAAACTACAGCGAAAACTAC<br>GCTGACGCAAGTTAA
7	pBAD-eGFP-AANDENYAANDENY	ATGCGGGGTTCTCATCATCATCATCATCATGGTATGGCTAGCATGACTGGTGGACAG<br>CAAATGGGTCGGGATCTGTACGAGAACCTGTACTTCCAGGGCTCGAGCATGGTGAGC<br>AAGGGCGAGGAGCTGTTCACCGGGGTGGTGCCCATCCTGGTCGAGCTGGACGGCGAC<br>GTAAACGGCCACAAGTTCAGCGTGTCCGGCGAGGGCGAGGGCGATGCCACCTACGGC<br>AAGCTGACCCTGAAGTTCATCTGCACCACCGGCAAGCTGCCCGTGCCCTGGCCCACC<br>CTCGTGACCACCCTGACCTACGGCGTGCAGTGCTTCAGCCGCTACCCCGACCACATG<br>AAGCAGCACGACTTCTTCAAGTCCGCCATGCCCGAAGGCTACGTCCAGGAGCGCACC<br>ATCTTCTTCAAGGACGACGGCAACTACAAGACCCGCGCCGAGGTGAAGTTCGAGGGC<br>GACACCCTGGTGAACCGCATCGAGCTGAAGGGCATCGACTTCAAGGAGGACGGCAAC<br>ATCCTGGGGCACAAGCTGGAGTACAACTACAACAGCCACAACGTCTATATCATGGCC<br>GACAAGCAGAAGAACGGCATCAAGGTGAACTTCAAGATCCGCCACAACATCGAGGAC<br>GGCAGCGTGCAGCTCGCCGACCACTACCAGCAGAACACCCCCATCGGCGACGGCCCC<br>GTGCTGCTGCCCGACAACCACTACCTGAGCACCCAGTCCGCCCTGAGCAAAGACCCC<br>AACGAGAAGCGCGATCACATGGTCCTGCTGGAGTTCGTGACCGCCGCCGGGATCACT<br>CTCGGCATGGACGAGCTGTACAAGGCTGCAAACGACGAAAACTACGCTGCAAACGAC<br>GAAAACTACTAA
8	pBAD-eGFP-AANDENYSENY	ATGCGGGGTTCTCATCATCATCATCATCATGGTATGGCTAGCATGACTGGTGGACAG<br>CAAATGGGTCGGGATCTGTACGAGAACCTGTACTTCCAGGGCTCGAGCATGGTGAGC<br>AAGGGCGAGGAGCTGTTCACCGGGGTGGTGCCCATCCTGGTCGAGCTGGACGGCGAC<br>GTAAACGGCCACAAGTTCAGCGTGTCCGGCGAGGGCGAGGGCGATGCCACCTACGGC<br>AAGCTGACCCTGAAGTTCATCTGCACCACCGGCAAGCTGCCCGTGCCCTGGCCCACC<br>CTCGTGACCACCCTGACCTACGGCGTGCAGTGCTTCAGCCGCTACCCCGACCACATG<br>AAGCAGCACGACTTCTTCAAGTCCGCCATGCCCGAAGGCTACGTCCAGGAGCGCACC<br>ATCTTCTTCAAGGACGACGGCAACTACAAGACCCGCGCCGAGGTGAAGTTCGAGGGC<br>GACACCCTGGTGAACCGCATCGAGCTGAAGGGCATCGACTTCAAGGAGGACGGCAAC<br>ATCCTGGGGCACAAGCTGGAGTACAACTACAACAGCCACAACGTCTATATCATGGCC<br>GACAAGCAGAAGAACGGCATCAAGGTGAACTTCAAGATCCGCCACAACATCGAGGAC<br>GGCAGCGTGCAGCTCGCCGACCACTACCAGCAGAACACCCCCATCGGCGACGGCCCC<br>GTGCTGCTGCCCGACAACCACTACCTGAGCACCCAGTCCGCCCTGAGCAAAGACCCC<br>AACGAGAAGCGCGATCACATGGTCCTGCTGGAGTTCGTGACCGCCGCCGGGATCACT<br>CTCGGCATGGACGAGCTGTACAAGGCTGCAAACGACGAAAACTACAGCGAAAACTAC<br>TAA
9	pBAD-eGFP-AANDENY	ATGCGGGGTTCTCATCATCATCATCATCATGGTATGGCTAGCATGACTGGTGGACAG<br>CAAATGGGTCGGGATCTGTACGAGAACCTGTACTTCCAGGGCTCGAGCATGGTGAGC<br>AAGGGCGAGGAGCTGTTCACCGGGGTGGTGCCCATCCTGGTCGAGCTGGACGGCGAC<br>GTAAACGGCCACAAGTTCAGCGTGTCCGGCGAGGGCGAGGGCGATGCCACCTACGGC<br>AAGCTGACCCTGAAGTTCATCTGCACCACCGGCAAGCTGCCCGTGCCCTGGCCCACC<br>CTCGTGACCACCCTGACCTACGGCGTGCAGTGCTTCAGCCGCTACCCCGACCACATG<br>AAGCAGCACGACTTCTTCAAGTCCGCCATGCCCGAAGGCTACGTCCAGGAGCGCACC<br>ATCTTCTTCAAGGACGACGGCAACTACAAGACCCGCGCCGAGGTGAAGTTCGAGGGC

		GACACCCTGGTGAACCGCATCGAGCTGAAGGGCATCGACTTCAAGGAGGACGGCAAC<br>ATCCTGGGGCACAAGCTGGAGTACAACTACAACAGCCACAACGTCTATATCATGGCC<br>GACAAGCAGAAGAACGGCATCAAGGTGAACTTCAAGATCCGCCACAACATCGAGGAC<br>GGCAGCGTGCAGCTCGCCGACCACTACCAGCAGAACACCCCCATCGGCGACGGCCCC<br>GTGCTGCTGCCCGACAACCACTACCTGAGCACCCAGTCCGCCCTGAGCAAAGACCCC<br>AACGAGAAGCGCGATCACATGGTCCTGCTGGAGTTCGTGACCGCCGCCGGGATCACT<br>CTCGGCATGGACGAGCTGTACAAGGCTGCAAACGACGAAAACTACTAA
10	pBAD-eGFP-AANDENYADAS	ATGCGGGGTTCTCATCATCATCATCATCATGGTATGGCTAGCATGACTGGTGGACAG<br>CAAATGGGTCGGGATCTGTACGAGAACCTGTACTTCCAGGGCTCGAGCATGGTGAGC<br>AAGGGCGAGGAGCTGTTCACCGGGGTGGTGCCCATCCTGGTCGAGCTGGACGGCGAC<br>GTAAACGGCCACAAGTTCAGCGTGTCCGGCGAGGGCGAGGGCGATGCCACCTACGGC<br>AAGCTGACCCTGAAGTTCATCTGCACCACCGGCAAGCTGCCCGTGCCCTGGCCCACC<br>CTCGTGACCACCCTGACCTACGGCGTGCAGTGCTTCAGCCGCTACCCCGACCACATG<br>AAGCAGCACGACTTCTTCAAGTCCGCCATGCCCGAAGGCTACGTCCAGGAGCGCACC<br>ATCTTCTTCAAGGACGACGGCAACTACAAGACCCGCGCCGAGGTGAAGTTCGAGGGC<br>GACACCCTGGTGAACCGCATCGAGCTGAAGGGCATCGACTTCAAGGAGGACGGCAAC<br>ATCCTGGGGCACAAGCTGGAGTACAACTACAACAGCCACAACGTCTATATCATGGCC<br>GACAAGCAGAAGAACGGCATCAAGGTGAACTTCAAGATCCGCCACAACATCGAGGAC<br>GGCAGCGTGCAGCTCGCCGACCACTACCAGCAGAACACCCCCATCGGCGACGGCCCC<br>GTGCTGCTGCCCGACAACCACTACCTGAGCACCCAGTCCGCCCTGAGCAAAGACCCC<br>AACGAGAAGCGCGATCACATGGTCCTGCTGGAGTTCGTGACCGCCGCCGGGATCACT<br>CTCGGCATGGACGAGCTGTACAAGAGCGAAAACTACGCTGACGCAAGTTAA
11	pBAD-eGFP-SENYALAA	ATGCGGGGTTCTCATCATCATCATCATCATGGTATGGCTAGCATGACTGGTGGACAG<br>CAAATGGGTCGGGATCTGTACGAGAACCTGTACTTCCAGGGCTCGAGCATGGTGAGC<br>AAGGGCGAGGAGCTGTTCACCGGGGTGGTGCCCATCCTGGTCGAGCTGGACGGCGAC<br>GTAAACGGCCACAAGTTCAGCGTGTCCGGCGAGGGCGAGGGCGATGCCACCTACGGC<br>AAGCTGACCCTGAAGTTCATCTGCACCACCGGCAAGCTGCCCGTGCCCTGGCCCACC<br>CTCGTGACCACCCTGACCTACGGCGTGCAGTGCTTCAGCCGCTACCCCGACCACATG<br>AAGCAGCACGACTTCTTCAAGTCCGCCATGCCCGAAGGCTACGTCCAGGAGCGCACC<br>ATCTTCTTCAAGGACGACGGCAACTACAAGACCCGCGCCGAGGTGAAGTTCGAGGGC<br>GACACCCTGGTGAACCGCATCGAGCTGAAGGGCATCGACTTCAAGGAGGACGGCAAC<br>ATCCTGGGGCACAAGCTGGAGTACAACTACAACAGCCACAACGTCTATATCATGGCC<br>GACAAGCAGAAGAACGGCATCAAGGTGAACTTCAAGATCCGCCACAACATCGAGGAC<br>GGCAGCGTGCAGCTCGCCGACCACTACCAGCAGAACACCCCCATCGGCGACGGCCCC<br>GTGCTGCTGCCCGACAACCACTACCTGAGCACCCAGTCCGCCCTGAGCAAAGACCCC<br>AACGAGAAGCGCGATCACATGGTCCTGCTGGAGTTCGTGACCGCCGCCGGGATCACT<br>CTCGGCATGGACGAGCTGTACAAGAGCGAAAACTACGCTTTAGCAGCTTAA
12	pBAD-eGFP-SENYADAS	ATGCGGGGTTCTCATCATCATCATCATCATGGTATGGCTAGCATGACTGGTGGACAG<br>CAAATGGGTCGGGATCTGTACGAGAACCTGTACTTCCAGGGCTCGAGCATGGTGAGC<br>AAGGGCGAGGAGCTGTTCACCGGGGTGGTGCCCATCCTGGTCGAGCTGGACGGCGAC<br>GTAAACGGCCACAAGTTCAGCGTGTCCGGCGAGGGCGAGGGCGATGCCACCTACGGC<br>AAGCTGACCCTGAAGTTCATCTGCACCACCGGCAAGCTGCCCGTGCCCTGGCCCACC<br>CTCGTGACCACCCTGACCTACGGCGTGCAGTGCTTCAGCCGCTACCCCGACCACATG<br>AAGCAGCACGACTTCTTCAAGTCCGCCATGCCCGAAGGCTACGTCCAGGAGCGCACC<br>ATCTTCTTCAAGGACGACGGCAACTACAAGACCCGCGCCGAGGTGAAGTTCGAGGGC<br>GACACCCTGGTGAACCGCATCGAGCTGAAGGGCATCGACTTCAAGGAGGACGGCAAC<br>ATCCTGGGGCACAAGCTGGAGTACAACTACAACAGCCACAACGTCTATATCATGGCC<br>GACAAGCAGAAGAACGGCATCAAGGTGAACTTCAAGATCCGCCACAACATCGAGGAC<br>GGCAGCGTGCAGCTCGCCGACCACTACCAGCAGAACACCCCCATCGGCGACGGCCCC<br>GTGCTGCTGCCCGACAACCACTACCTGAGCACCCAGTCCGCCCTGAGCAAAGACCCC<br>AACGAGAAGCGCGATCACATGGTCCTGCTGGAGTTCGTGACCGCCGCCGGGATCACT<br>CTCGGCATGGACGAGCTGTACAAGAGCGAAAACTACGCTGACGCAAGTTAA

13	pBAD-eGFP-ALAA	ATGCGGGGTTCTCATCATCATCATCATCATGGTATGGCTAGCATGACTGGTGGACAG<br>CAAATGGGTCGGGATCTGTACGAGAACCTGTACTTCCAGGGCTCGAGCATGGTGAGC<br>AAGGGCGAGGAGCTGTTCACCGGGGTGGTGCCCATCCTGGTCGAGCTGGACGGCGAC<br>GTAAACGGCCACAAGTTCAGCGTGTCCGGCGAGGGCGAGGGCGATGCCACCTACGGC<br>AAGCTGACCCTGAAGTTCATCTGCACCACCGGCAAGCTGCCCGTGCCCTGGCCCACC<br>CTCGTGACCACCCTGACCTACGGCGTGCAGTGCTTCAGCCGCTACCCCGACCACATG<br>AAGCAGCACGACTTCTTCAAGTCCGCCATGCCCGAAGGCTACGTCCAGGAGCGCACC<br>ATCTTCTTCAAGGACGACGGCAACTACAAGACCCGCGCCGAGGTGAAGTTCGAGGGC<br>GACACCCTGGTGAACCGCATCGAGCTGAAGGGCATCGACTTCAAGGAGGACGGCAAC<br>ATCCTGGGGCACAAGCTGGAGTACAACTACAACAGCCACAACGTCTATATCATGGCC<br>GACAAGCAGAAGAACGGCATCAAGGTGAACTTCAAGATCCGCCACAACATCGAGGAC<br>GGCAGCGTGCAGCTCGCCGACCACTACCAGCAGAACACCCCCATCGGCGACGGCCCC<br>GTGCTGCTGCCCGACAACCACTACCTGAGCACCCAGTCCGCCCTGAGCAAAGACCCC<br>AACGAGAAGCGCGATCACATGGTCCTGCTGGAGTTCGTGACCGCCGCCGGGATCACT<br>CTCGGCATGGACGAGCTGTACAAGGCTTTAGCAGCTTAA
14	pBAD-eGFP-ADAS	ATGCGGGGTTCTCATCATCATCATCATCATGGTATGGCTAGCATGACTGGTGGACAG<br>CAAATGGGTCGGGATCTGTACGAGAACCTGTACTTCCAGGGCTCGAGCATGGTGAGC<br>AAGGGCGAGGAGCTGTTCACCGGGGTGGTGCCCATCCTGGTCGAGCTGGACGGCGAC<br>GTAAACGGCCACAAGTTCAGCGTGTCCGGCGAGGGCGAGGGCGATGCCACCTACGGC<br>AAGCTGACCCTGAAGTTCATCTGCACCACCGGCAAGCTGCCCGTGCCCTGGCCCACC<br>CTCGTGACCACCCTGACCTACGGCGTGCAGTGCTTCAGCCGCTACCCCGACCACATG<br>AAGCAGCACGACTTCTTCAAGTCCGCCATGCCCGAAGGCTACGTCCAGGAGCGCACC<br>ATCTTCTTCAAGGACGACGGCAACTACAAGACCCGCGCCGAGGTGAAGTTCGAGGGC<br>GACACCCTGGTGAACCGCATCGAGCTGAAGGGCATCGACTTCAAGGAGGACGGCAAC<br>ATCCTGGGGCACAAGCTGGAGTACAACTACAACAGCCACAACGTCTATATCATGGCC<br>GACAAGCAGAAGAACGGCATCAAGGTGAACTTCAAGATCCGCCACAACATCGAGGAC<br>GGCAGCGTGCAGCTCGCCGACCACTACCAGCAGAACACCCCCATCGGCGACGGCCCC<br>GTGCTGCTGCCCGACAACCACTACCTGAGCACCCAGTCCGCCCTGAGCAAAGACCCC<br>AACGAGAAGCGCGATCACATGGTCCTGCTGGAGTTCGTGACCGCCGCCGGGATCACT<br>CTCGGCATGGACGAGCTGTACAAGGCTGACGCAAGTTAA
