# Supplementary material for: A Uniform Benchmark for Testing SsrA-Derived Degrons in the Escherichia coli ClpXP Degradation Pathway
Source: Molecules. 2021 Sep 30;26(19):5936. doi: 10.3390/molecules26195936 (PMC8512704; doi:10.3390/molecules26195936)
Supplement: Supplementary file 1 [file molecules-26-05936-s001.zip › molecules-1396538-supplementary/Supplemetary Table S2.pdf]

Supplemetary material

A uniform benchmark for testing ssrA-derived degrons in the Escherichia coli ClpXP degradation pathway

Maria Magdalena Klimecka, Anna Antosiewicz, Matylda Anna Izert, Patrycja Emanuela Szybowska, Piotr Krzysztof Twardowski, Clara Delaunay and Maria Wiktoria Górna

Supplemetary Table S2: List of primers used for cloning the protease complex components and site-directed mutagenesis for preparing pBAD-eGFP-degron constructs

	Primer name	Primer sequence (5′→ 3′)
1	ClpX-pET28a For	TGATTGAAGTCTACCAGGAACAAACCGGTGGATCCATGACAGATAAACGCAAAGATG
2	ClpX-pET28a Rev	CGGATCTCAGTGGTGGTGGTGGTGGTGCTCGAGTTATTCACCAGATGCCTGTTGCG
3	ClpX-pBAD For	TTTGTTTAACTTTAAGAAGGAGATATACATATGGGCAGCAGCCATCA
4	ClpX-pBAD Rev	CTTCTCTCATCCGCCAAAACAGCCAAGCTTTTATTCACCAGATGCCTGTTGCG
5	ClpP-pET28a For	TGATTGAAGTCTACCAGGAACAAACCGGTGGATCCATGTCATACAGCGGCGAACG
6	ClpP-pET28a Rev	CGGATCTCAGTGGTGGTGGTGGTGGTGCTCGAGTCAATTACGATGGGTCAGAATCGAATCG
7	SspB-pET28a For	TGATTGAAGTCTACCAGGAACAAACCGGTGGATCCATGGATTTGTCACAGCTAACACCACG
8	SspB-pET28a Rev	CGGATCTCAGTGGTGGTGGTGGTGGTGCTCGAGTTACTTCACAACGCGTAATGCCGGTCG
9	eGFP-AANDENYALAA-pBAD For	TACGCTTTAGCAGCTTAAGAATTCGAAGCTTGGCTG
10	eGFP-AANDENYALAA-pBAD Rev	GTTTTCGTCGTTTGCAGCCTTGTACAGCTCGTCCATG
11	eGFP-AANDENYSENYALAA-pBAD For	CGAAAACTACGCTTTAGCAGCTTAAGAATTCGAAGCTTGGC
12	eGFP-AANDENYSENYALAA-pBAD Rev	CTGTAGTTTTCGTCGTTTGCAGCCTTGTACAGCTCGTCCATG
13	eGFP-AANDENYSENYADAS-pBAD For	CTACGCTGACGCAAGTTAAGAATTCGAAGCTTGGC
14	eGFP-AANDENYSENYADAS-pBAD Rev	TTTTCGTCGTTTGCAGCCTTGTACAGCTCGTCCATG
15	eGFP-AANDENYAANDENY-pBAD For	CTACAGCGAAAACTACTAAGAATTCGAAGCTTGGC
16	eGFP-AANDENYAANDENY-pBAD Rev	TTTTCGTCGTTTGCAGCCTTGTACAGCTCGTCCATG
17	eGFP-AANDENYSENY-pBAD For	GCTTTAGCAGCTTAAGAATTCGAAGCTTGGC
18	eGFP-AANDENYSENY-pBAD Rev	TTTTCGTCGTTTGCAGCCTTGTACAGCTCGTCCATG
19	eGFP-AANDENY-pBAD For	GAAAACTACTAAGAATTCGAAGCTTGGCTG

20	eGFP-AANDENY-pBAD Rev	GTCGTTTGCAGCCTTGTACAGCTCGTCCAT
21	eGFP-AANDENYADAS-pBAD For	CTACAGCGAAAACTACTAAGAATTCGAAGCTTGGC
22	eGFP-AANDENYADAS-pBAD Rev	TTTTCGTCGTTTGCAGCCTTGTACAGCTCGTCCATG
23	eGFP-SENYALAA-pBAD For	GCTTTAGCAGCTTAAGAATTCGAAGCTTGGC
24	eGFP-SENYADAS-pBAD For	GCTGACGCAAGTTAAGAATTCGAAGCTTGGC
25	eGFP-SENYALAA/ADAS-pBAD Rev	GTAGTTTTCGCTCTTGTACAGCTCGTCCATG
26	eGFP-ALAA-pBAD For	GCAGCTTAAGAATTCGAAGCTTGGC
27	eGFP-ALAA-pBAD Rev	TAAAGCCTTGTACAGCTCGTCCATG
28	eGFP-ADAS-pBAD For	GCAAGTTAAGAATTCGAAGCTTGGC
29	eGFP-ADAS-pBAD Rev	GTCAGCCTTGTACAGCTCGTCCATG
